# Guidelines for Experiments Using Antisense Oligonucleotides and Double-Stranded RNAs

**DOI:** 10.1089/nat.2018.0772

**Published:** 2019-05-30

**Authors:** Keith T. Gagnon, David R. Corey

**Affiliations:** ^1^ Department of Biochemistry and Molecular Biology, School of Medicine, Southern Illinois University, Carbondale, Illinois.; ^2^ Department of Chemistry and Biochemistry, Southern Illinois University, Carbondale, Illinois.; ^3^ Departments of Pharmacology and Biochemistry, UT Southwestern Medical Center at Dallas, Dallas, Texas.

**Keywords:** antisense oligonucleotide, duplex RNA, controls, guidelines

## Abstract

After decades of research and development, synthetic nucleic acids are beginning to enjoy significant success in the clinic. Approved drugs have increased interest in the field, and many basic research studies have focused on synthetic nucleic acids to control the action of messenger RNA and noncoding RNAs. Unfortunately, experimental designs are often inadequate, resulting in misleading interpretation of data and unconvincing work that wastes resources and does little to advance the field. The goal of this commentary is to outline the problems facing many researchers, especially those new to the use of synthetic oligonucleotides. We describe the minimum control experiments necessary to build a strong case for real effects that are likely due to interactions at the intended molecular target. A common set of standards for preparing and judging experiments should facilitate better interpretation of data and publications that contribute positively to using synthetic nucleic acids as tools and drugs.

## Introduction

Generality is the great strength of synthetic nucleic acids as agents to control gene expression [1]. It is straightforward to synthesize oligonucleotides complementary to a target RNA transcript and to design panels of oligonucleotides for screening to identify the most potent agents. The concept behind using synthetic oligonucleotides is so clear that investigators are often tempted to conclude that a complementary synthetic nucleic acid will find its RNA target, modulate gene expression, and elicit the desired phenotype.

**FIG. 1.**
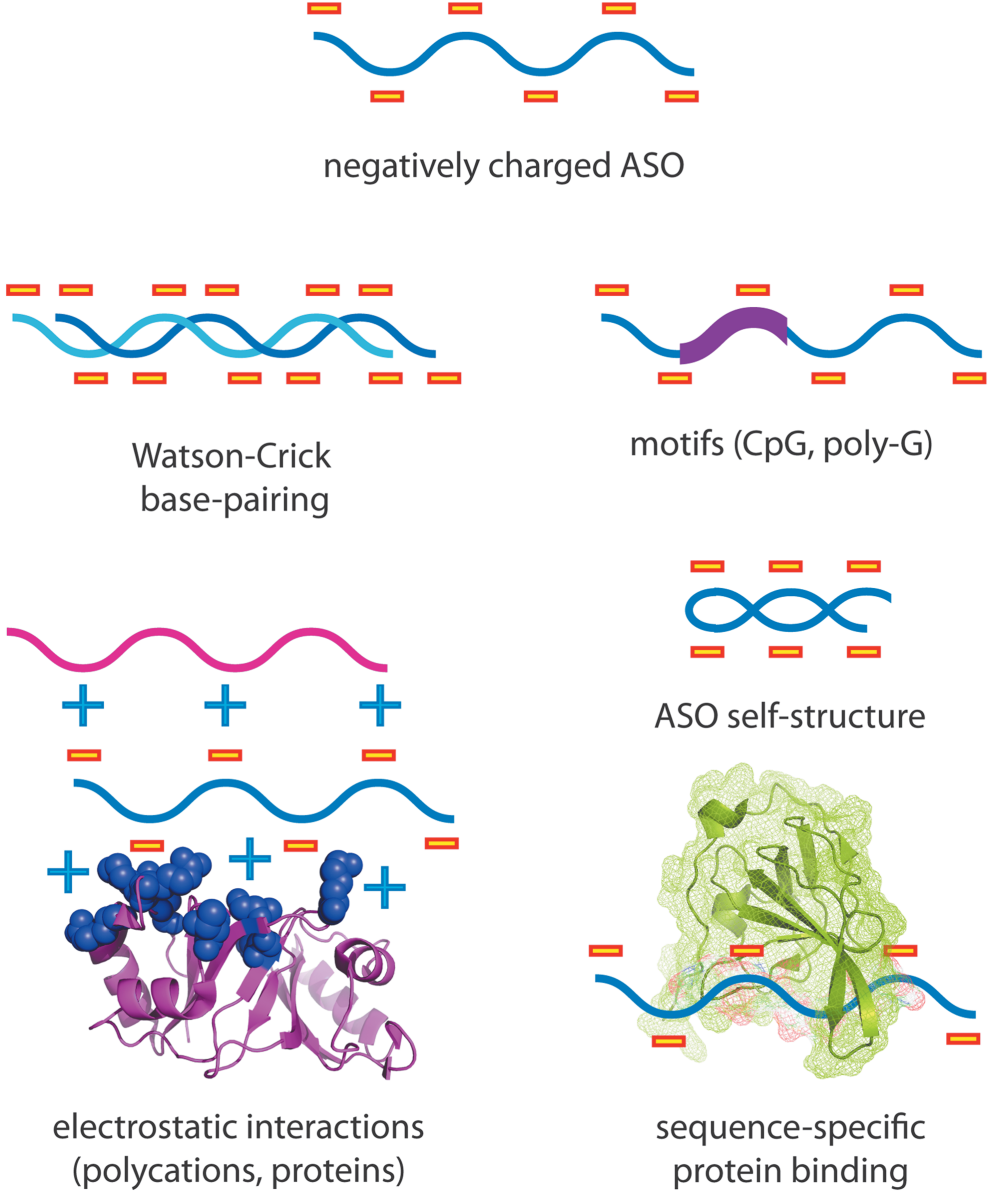
Molecular interactions of synthetic oligonucleotides. Synthetic oligonucleotides, such as ASOs, can mediate both specific and nonspecific molecular interactions. Their multivalent negative charge, base-pairing potential, and sequence motifs can be responsible for various forms of nonspecific interactions and off-target effects. ASOs, antisense oligonucleotides.

The flaw in this reasoning is that oligonucleotides are relatively large amphipathic compounds that form many interactions. They possess a highly negatively charged backbone on one face and hydrophobic bases possessing the potential to pair with other nucleic acids on the other. While oligonucleotides undergo sequence specific hybridization with other nucleic acids, they also have the potential to form undesired interactions, both inside and outside of cells. These include electrostatic interactions with polycations and positively charged proteins, Watson-Crick and noncanonical base-pairing with themselves and other nucleic acids, and sequence-specific interaction with proteins (Fig. 1).

Of course, all small molecules, antibodies, and other starting points for drug discovery confront this problem of “off-target” effects, which potentially confounds experimental interpretation. The special pitfall for scientists using synthetic oligonucleotides lies in the ease of access and the deceptively simple Watson-Crick base-pairing rules.

Because the rules for complementary recognition are so predictable, researchers expect that base-pairing to the target sequence will be the predominant interaction and the most likely explanation for the resulting phenotype. When the expected gene expression does change, or the expected phenotype is observed, investigators jump to the obvious conclusion that the experiment has worked as planned. They may move on to the next experiment, never realizing that the key experimental foundation to future research was built on shifting sand.

The potential for antisense oligonucleotides (ASOs) to produce off-target effects has been known for decades. Since the 1990s, commentaries similar in intent to this one have pointed out the danger, illustrated the widespread nature of the problem, and described proper controls and experimental considerations [2–9]. These commentaries contain detailed explanations for the origins of confounding effects that are as valid today as they were 20 years ago. Those details will not be repeated here, but these older publications merit close examination by new generations of investigators. Similar warnings regarding the use and misuse of duplex RNAs were published soon after mammalian RNA interference (RNAi) was first described in 2001 [10].

Despite this long history of warnings, investigators continue to use synthetic oligonucleotides improperly. On the surface, publications may appear to be thorough and often describe a remarkable array of experiments, starting with target identification and finishing with modulation of *in vivo* physiology. When the figures are examined in detail, however, they often fail to provide convincing evidence that observations result from “on target” interactions at the intended RNA sequence. This outcome is not unique to articles describing ASOs or duplex RNAs and fits into the broader problem of a scientific culture that prioritizes the creation of grand research reports that fit the mold set by high profile journals [11].

The problem of improper use of oligonucleotides is growing. More investigators are being attracted to the field because of recent Food and Drug Administration approvals and increased interest in RNA biology, particularly long noncoding RNAs (lncRNAs) and microRNAs (miRNAs). These investigators are unfamiliar with the relatively old literature counseling caution [2–10] and may lack an appreciation for the challenges encountered when using oligonucleotides.

**FIG. 2.**
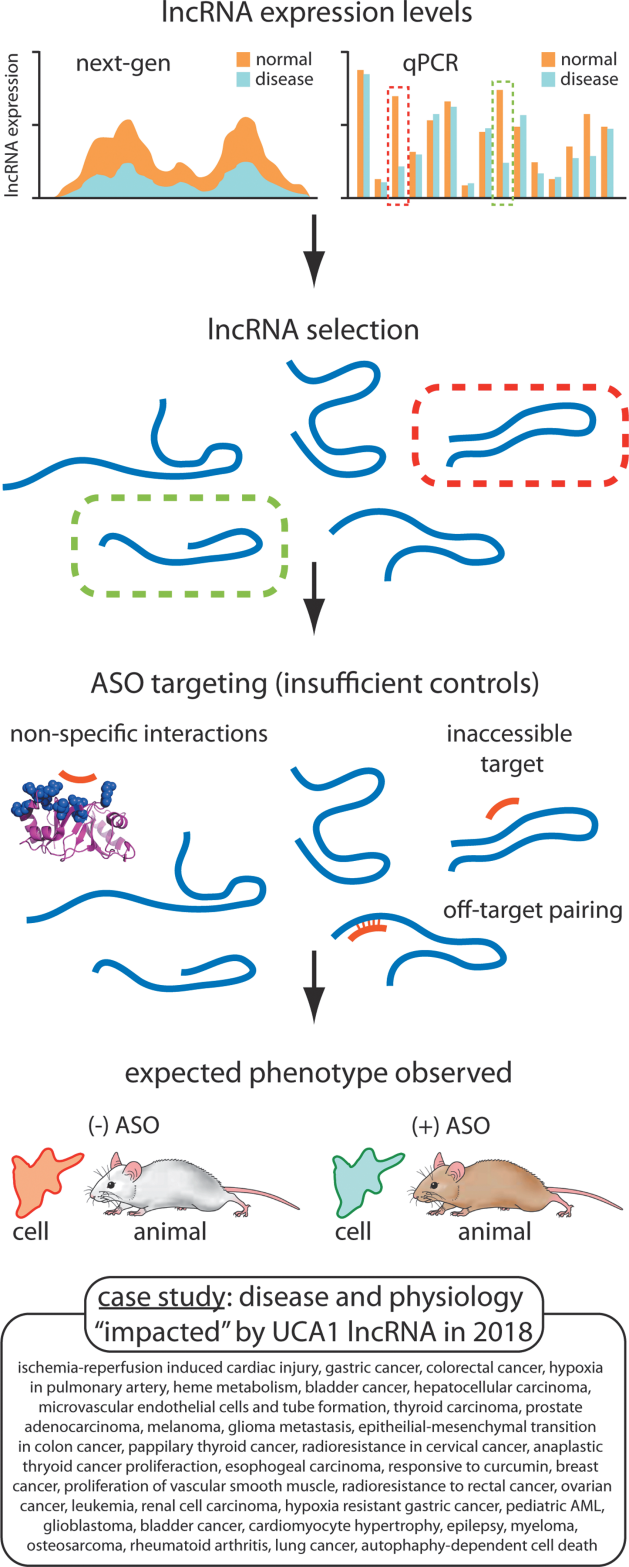
Investigation of lncRNA mechanism has triggered renewed use, and misuse, of synthetic oligonucleotides. Selection of lncRNAs for investigation often begins with gene expression data, followed by synthetic oligonucleotides or short hairpin RNAs to knockdown the lncRNA. However, deficient experimental design can lead to observation of phenotypes that may not be connected to a molecular mechanism involving the targeted lncRNA. One lncRNA that has been the subject of such studies, UCA1, has been implicated in a growing number of physiological and disease pathways. lncRNA, long noncoding RNA; qPCR, quantitative PCR; UCA1, urothelial carcinoma associated.

The mechanisms of action of miRNAs and lncRNAs are often obscure, increasing the need for meticulous planning and experimentation. Articles focusing on lncRNAs or miRNAs often begin by selecting a specific noncoding RNA (ncRNA) as the focus, often without strong rationale (Fig. 2). These articles usually emphasize descriptive studies of phenotype over investigation into molecular mechanisms.

We offer one area of investigation as an example. The urothelial carcinoma associated (*UCA1*) gene was first identified as a ncRNA in 2006 [12]. As of late 2018, 242 publications have appeared citing UCA1. For just the first 10 months of 2018, over fifty studies appeared implicating UCA1 in three dozen different types of cancer, other diseases, or normal physiological processes. Eighteen different miRNAs were reported to be associated with UCA1. Many for these studies used duplex RNAs like short hairpin RNAs (shRNAs), small interfering RNAs (siRNAs), or miRNA mimics.

While we make no judgments about the value of these peer-reviewed publications, none of the studies fully adhered to the minimum standards for controls outlined below. It is difficult to believe that a single ncRNA could perform so many roles or be regulated by so many miRNAs. The proliferation of inadequately controlled publications leads to confusion, obscures the contributions of better-designed projects, and makes it difficult to build a reliable foundation for future work.

This commentary aims to provide practical advice for performing experiments with synthetic nucleic acids and to outline minimum standards for published research (Table 1). Our guidelines are designed to assist investigators to efficiently use resources to produce persuasive research and help the community critically assess articles that use oligonucleotides to control gene expression.

**Table 1.** Summary of Minimum Guidelines for Peer-Reviewed Studies Using Synthetic Nucleic Acids to Control Gene Expression

Overall
Clear concise arguments written to inform a skeptical reader
General textual requirements
The source of a hypothesis, data that argue against “cherry picking” a hypothesis, and selection criteria must be clearly presented
The evidence suggesting that observed effects are the result of “on target” engagement must be plainly presented
Experimental limitations and uncertainty must be candidly presented
Potential alternative hypotheses that might explain results but do not involve “on-target” engagement must be acknowledged
Cell culture
Two independent silencing duplex RNAs or antisense oligonucleotides
One multiple mismatch-containing control
One scrambled control
Animal models
Robust and thorough cell culture experiments
One mismatched or scrambled control
More controls/supporting experiments may be needed for antiproliferative phenotypes

## General Experimental Design

Experiments with synthetic oligonucleotides, like siRNAs and ASOs, should meet all the standards that any good experiment is expected to meet [13–15]. Experiments should be repeated multiple times to ensure reproducibility. If gene knockdown is quantified, the number of replicates should be sufficient to produce statistically useful results. If a result is both remarkable and unexpected, it might be wise to have it be repeated by a separate set of hands in the laboratory or even replicated in another laboratory.

All experiments start with a hypothesis. Many projects that use modern methods to collect large amounts of data risk “cherry picking” results to fit a hypothesis and justify focusing on a particular messenger RNA (mRNA), ncRNA, miRNA, or RNA-associated protein partner. Transparent, replicated, and robust data should be presented to justify why the starting point stands out from the mass of other data collected by techniques like next-generation RNA sequencing or mass spectrometry.

There is always a trade-off between experiments that are designed to be as definitive as possible and the resources available to a laboratory. We recognize that no experiment investigating a complex biological process produces an outcome that is completely proven. There will always be some uncertainty. Uncertainty, however, should not be compounded by poor planning, execution, and interpretation of experiments involving synthetic nucleic acids.

## Measuring Gene Expression

In many cases the desired outcome is a change in RNA levels. RNA levels are commonly detected using reverse transcription quantitative PCR (RT-qPCR) for the specific target RNA. Although RT-qPCR is a valuable technique that should be used routinely, the numerical output is indirect and potentially misleading if not examined carefully.

It is important to realize the limitations of PCR and to always abide by recognized standards when obtaining, interpreting, and presenting data [16,17]. For example, identifying a “housekeeping” gene that does not change its expression after treatment is sometimes a frustrating process, but must be done with care and transparency. While time-consuming to perform, northern blot directly measures RNA levels and reveals the size of the RNA on a gel.

For monitoring many transcripts at one time, more modern techniques like next-generation sequencing are used. Sequencing techniques are also indirect and require precise normalization protocols and should be used only after more specific methods to validate siRNA or ASO function are performed. A complete description of the care needed for these experiments would likely require a separate commentary. Nonetheless, global changes in gene expression at different stages after synthetic oligonucleotide treatment help build a case for activation of expected pathways or identifying unsuspected off-target effects.

When targeting RNA, a change in protein levels is often the desired outcome. In these cases, western analysis provides convincing evidence because the results are primary data—they directly reveal protein molecular weight and relative expression level. The quality of data is obvious and offers insight into the care with which experiments are performed. If the antibody used for protein detection has not been previously characterized, it is usually necessary to validate its ability to detect the target protein. Other analytical methods are becoming available to quantify specific protein levels, but western blot remains the gold standard.

Rigorous evaluation should include dose–response curves. Dose–response curves are useful because calculation of an inhibitory concentration (IC_50_) or effective concentration (EC_50_) when expression is increased or when splicing changes provides direct comparisons between experiments. By also monitoring cell viability or toxicity over a titration course, investigators determine a window for safe and effective use of nucleic acids.

More generally, the multiple points of a dose–response curve support one another and increase confidence in the reproducibility of the experiments. Dose–response curves convey a message to readers that the system is reproducible enough to allow multiple experiments at different concentrations that produce a consistent body of data. By contrast, reports that offer only a few examples of gene expression changes invite skepticism.

## Measuring Cell Uptake

Many researchers use microscopy to follow oligonucleotide uptake by cells, both in cell culture and *in vivo*. Microscopy is often a useful adjunct experiment. However, unless researchers are studying the mechanism of oligonucleotide uptake, the goal of most experiments is modulation of gene expression. If target gene expression changes, the cellular localization of the silencing agent was probably appropriate.

Microscopy can also yield confounding results about localization. The presence of a fluorescent tag may affect localization and make interpretations uncertain. The use of fixatives rather than live cells may alter localization of tagged molecules [18]. There is a danger that a fluorescent tag, especially one at the 3′ or 5′ termini, may detach. Experimenters should justify why observation of fluorescence is a valid marker of oligonucleotide localization.

Microscopy may be more valuable *in vivo* when experimenters are attempting to demonstrate whether an oligonucleotide is entering tissue. It is also possible to evaluate tissue uptake using antibodies that recognize a unique nucleic acid chemistry or conjugate, such as phosphorothioate backbone linkages or biotin tags [19]. Even in these cases, demonstration that the expression of a target gene is being reduced through the expected biochemical mechanism would be more exciting. For example, 5′ reverse amplification of cDNA ends (5′-RACE) is a technique that allows detection of the exact cleavage sites induced by a synthetic nucleic acid. 5′-RACE does not provide a quantitative determination of the efficiency of gene knockdown, but was used to support one of the earliest reports that duplex RNA silenced a therapeutic target gene *in vivo* [20].

## Controls: Cell Culture

**FIG. 3.**
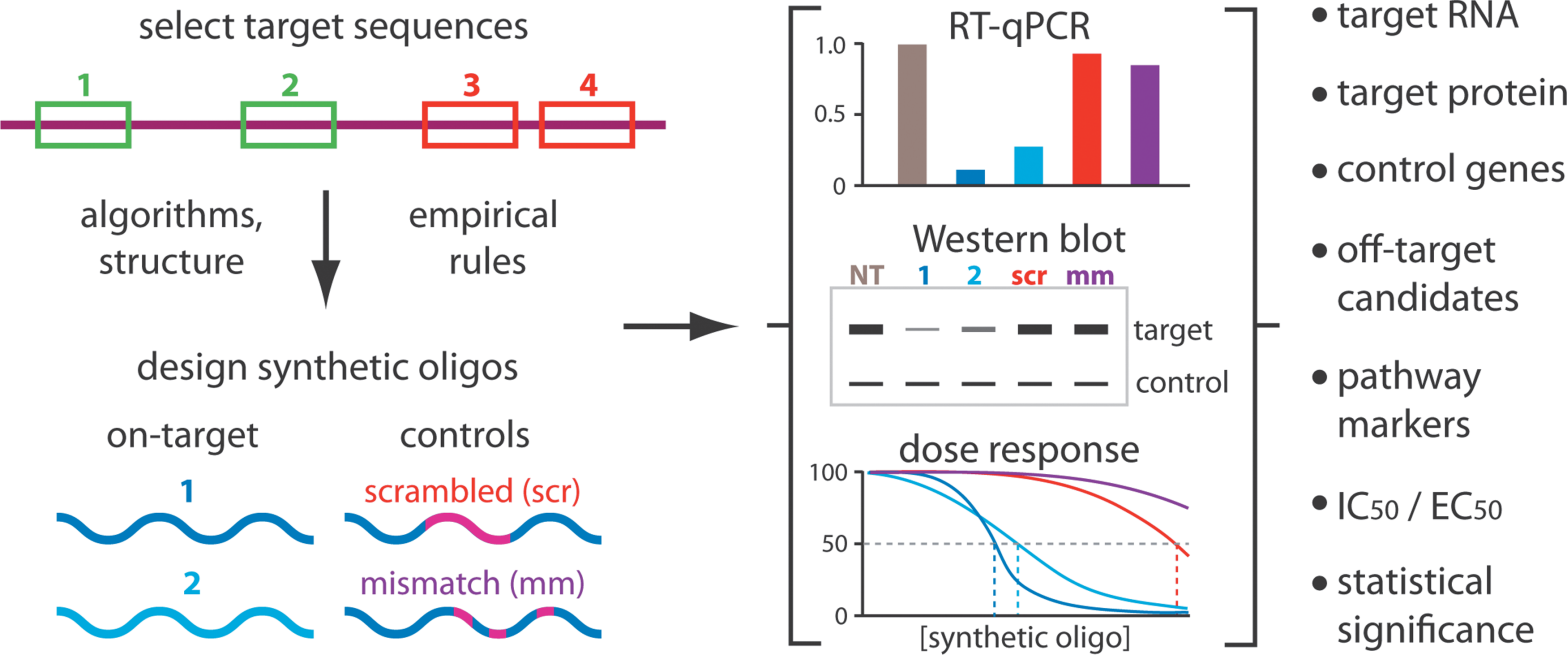
General guidelines for performing convincing gene expression knockdown experiments with synthetic oligonucleotides. Once a specific target RNA has been identified, several putative target sequences are chosen using empirical rules (such as preferred target regions, desired experimental outcome, and chemical modification limitations), algorithms to predict the best sites (with minimal off targets), and available structural information (to improve accessibility). At least two on-target oligonucleotides and two control oligonucleotides should be designed. Controls should be at least one scrambled and one mismatch oligonucleotide based on the lead on-target oligonucleotide. Titrations should be performed to obtain dose–response curves. RNA levels and protein levels (when applicable) should be measured. EC_50_, effective concentration; IC_50_, inhibitory concentration; RT-qPCR, reverse transcription quantitative PCR.

One of the great strengths of experiments that use synthetic oligonucleotides to silence gene expression is the ease with which controls may be obtained and tested (Fig. 3). It is possible to precisely tailor negative controls by changing the nucleotide position at any location.

Surprisingly, although controls are easy to obtain, they are often underused. Many experiments test just one gene silencing agent and one control. The problem with this experimental plan is that either the intended gene silencing agent or the control may exert off-target effects. Since one is normalized to the other, off-target issues may go unnoticed and dramatically alter the interpretation of the experiment.

At a minimum, experiments designed to break new ground should include an oligonucleotide that has three or four mismatches relative to the target sequence. If an RNAi mechanism is used, one or two of those mismatches should be in the potential seed region. The goal of these mismatches is to substantially reduce the affinity of the ASO for the target sequence or disrupt the siRNA guide strand's seed sequence complementarity and reduce potential miRNA-like mechanisms of gene regulation.

A second, “scrambled” control should also be used. Scrambled controls have the same percentage nucleotide composition as an active complementary ASO or duplex RNA, but contain blocks of bases that are switched in position. Scrambled designs preserve, to the greatest extent possible, neighboring base sequences. Short sequences, most famously CpG [21] and G-tracts [22], exert off-target effects independent of the overall oligonucleotide sequence. Toxicity in animals can also be associated with specific sequence motifs [23].

The possibility that ASOs or duplex RNAs complementary to the target may also be exerting confounding off-target effects should be addressed. A simple method for achieving this is to synthesize and test a second ASO or duplex RNA that is complementary to another sequence within the target RNA. If two different complementary oligonucleotides produce the same effect on target gene expression and cell phenotype, and mismatch or scrambled controls do not produce that result, it is reasonable to conclude that the observations are on-target effects.

The use of two on-target oligonucleotides and two control oligonucleotides is a minimum requirement (Fig. 3). The use of additional on-target or control oligonucleotides will always strengthen an experiment. To our knowledge, no article has ever been rejected for having too many controls. Use of additional compounds is especially important if the differences between gene expressions before and after compound addition are small, or if the observed cellular phenotype is subtle.

Controls become a dominant consideration when applications go beyond the routine. For example, many investigators are interested in the function of ncRNAs in cell nuclei and use siRNAs or shRNAs to modulate their expression. It is known that protein RNAi factors exist in cell nuclei and that nuclear activities are observable [24]. RNAi, however, is more reliable for cleavage of cytoplasmic targets relative to nuclear ones [25]. Experiments that use duplex RNAs in the nucleus, therefore, require special care and well-justified controls.

Given the importance of proper controls, it would be helpful if suppliers that sell oligonucleotides adopt policies that encourage the purchase of a suite of oligonucleotides for testing gene knockdown at an intended target. Setting a high price per oligonucleotide discourages proper use of controls, likely limits the success of projects, and reduces the long-term market for chemically modified oligonucleotides. Pricing that encourages good experiments would likely benefit both researchers and suppliers.

Controls must be similar in length and chemical composition to ASOs that are proposed to be acting through an “on target” mechanism. For example, a locked nucleic acid ASO would be a poor control for an active compound that contains 2′-*O*-methyl nucleotides. A 21 nucleotide ASO would be a poor control for an 18 nucleotide compound.

Many reports compare the effects of “on target” ASOs or duplex RNAs with controls where either lipid is used in the absence of oligonucleotide or where cells are untreated. Neither of these are particularly helpful controls. In general, the combination of lipid and oligonucleotide is much more likely to produce off-target effects than lipid alone or oligonucleotide alone. In cell culture, cell death is the most obvious effect. The effort used to analyze “mock” or “transfection reagent only” controls is probably better spent testing additional positive control or negative control ASOs or duplex RNAs.

Gymnotic delivery offers an alternative to lipid-mediated delivery for ASOs. In this approach, “naked” ASOs containing phosphorothioate backbone linkages in saline are added directly to cells [26]. This approach is advantageous because it is simple and avoids the potential for toxicity associated with the combination of lipid and ASO. The disadvantages are that the approach requires a higher concentration of ASO and may not be suitable for some cell lines. The need to use well-designed control oligonucleotides is similar whether lipid-mediated or gymnotic delivery is used.

## Controls: Animal experiments

In principle, the control experiments required for projects that test ASOs and duplex RNAs in animals are the same as those used in cell culture. The more controls used, the more persuasive the outcome. However, we recognize that it is more expensive to obtain the amount of oligonucleotide necessary to dose animals, especially if dosing needs to be done repeatedly.

A full suite of controls is ideal. However, it is also acceptable to thoroughly test ASOs and siRNAs in cell culture before animal experiments and to clearly present these experiments within the article text. The culture cells should be as similar as possible to the relevant target animal tissue. The animal experiments may then use one fully complementary, on-target oligonucleotide and one noncomplementary control (preferably a validated mismatch).

Researchers should use an ample number of animals and avoid experiments that are underpowered—all animals are sacrificed needlessly if the numbers used are too small to be statistically convincing. Whenever possible, gene knockdown should be evaluated in target tissues for target and control genes. If possible, secondary physiologic endpoints should also be monitored. The need to use sufficient animals to achieve convincing results should be consistent with experimental design principles of “replace, reduce, and refine” to optimize animal welfare and return value for their use [27].

As of 2019, gene knockdown in animal models is being routinely achieved in the liver and, to a lesser extent for ASOs, the central nervous system [1]. Success in other organs is less consistent. When novel genes are being targeted in extrahepatic tissues, a higher standard of caution should be used when interpreting experiments.

For example, an experimenter claiming efficient gene knockdown in the heart should realize that the result goes against current dogma. Challenging dogmas can lead to breakthroughs, but great care should be taken when building a case that observed effects are due to on-target interactions in the tissues of interest and not systemic off-target effects in other tissues. Readers should be most skeptical when gene knockdown is claimed in extrahepatic tissue and should search for persuasive supporting data.

## Special Case: Antiproliferative Oligonucleotides

The goal of many experiments in cancer research is to identify compounds that will reduce tumor cell proliferation. This paradigm poses a special problem for oligonucleotide therapeutics since all oligonucleotides have the potential to cause cell toxicity when used at high enough concentrations. Since oligonucleotides are often assumed to regulate their intended targets, the observed toxic effects are presumed to be the antiproliferative phenotype expected by the researcher.

When cell proliferation is affected the expression of many genes will change. Therefore, monitoring expression of the target gene has the potential to be misleading because changes may be linked to global expression changes rather than an on-target interaction. Unfortunately, these problems have led to mechanistic uncertainties that persist through advanced clinical trials and reduce the likelihood of success [28].

The best method to build a case for on-target effects is to strictly adhere to the guidelines governing positive and negative controls. At least two different “on-target” oligonucleotides should produce the observed antiproliferative phenotype. Multiple mismatch-containing and scrambled oligonucleotides should leave proliferation unaffected. Because antiproliferative phenotypes are difficult to validate, using multiple well-designed controls in animal experiments is essential even though these will increase the expense of the experiment.

## Special Case: Alteration of Splicing

These guidelines have focused on oligonucleotides that increase or decrease expression of a specific mRNA. There are also several examples of ASOs that successfully alter gene splicing [1]. In these experiments the addition of an ASO shifts transcript production from one splice variant to another. When examining transcript production, it is unlikely that this shift would occur because of an artifact. Nevertheless, a mismatch-containing and scrambled ASO should be used to rule out an outcome in which the transfection is influencing splicing.

For many experiments using ASOs to affect splicing, the final goal will be alteration of a cellular function. Alteration of function is much more susceptible to artifacts than is the change of splicing pattern for a targeted gene. Therefore, the minimum controls needed for anti-splicing ASOs and ASOs intended to up- or downregulate expression of a mRNA are similar—at least one mismatch and one scrambled oligonucleotide should be used for cell culture experiments.

## Minimum Common Requirements for Diverse Applications

Nucleic acids that control gene expression have many different types of targets and function by different mechanisms. Types of targets include mRNA, pre-mRNA, lncRNAs, and miRNAs. They function through recruiting RNAse H, by acting through the RNA induced silencing complex, by blocking a miRNA, by mimicking a miRNA, or by blocking splicing. These are not inclusive lists, and we anticipate that RNA biology will continue to surprise investigators with novel cellular targets and mechanisms of action.

## Roles of Authors, Reviewers, Editors, and Readers

These guidelines cannot anticipate every situation that will be faced by authors, editors, and reviewers. It is essential that authors describe how rigorous controls are built into their experimental plan and provide arguments for why their results should be considered robust. Ideally, authors should complement demonstrations of gene knockdown with thorough biochemical and mechanistic analysis. The extent to which supporting biochemical data supports conclusions should be clearly described.

Why should the reader believe that a change in gene expression is not an off-target effect? Why should the reader believe that physiologic consequences are not off-target effects? Why should the reader believe that intracellular localization or *in vivo* localization is not an artifact of the method used for visualization? The burden is on authors to foresee objections and supply answers. Regardless of the exact nature of the application, the burden is on editors, reviewers, and readers to demand that this information be supplied.

## Conclusion

ASOs and duplex RNAs have demonstrated their potential as experimental tools and life-saving drugs. As their success increases their use, it becomes even more important to remember the lessons of the past [2–10]. Researchers should take the responsibility of being their own greatest skeptic. They should clearly describe the experimental evidence that leads them to conclude that observed effects are likely due to the engagement of their synthetic oligonucleotide with the intended, complementary target sequence.

Transparency and candor are essential. Readers should be skeptical and insist that data be persuasive. Authors must design their experiments to be robust and explain the strengths of their experimental design in sufficient detail to persuade readers that results provide a firm foundation for future progress.

All fields of biologic science face challenges when striving to achieve robust and reproducible results [11,13–15]. There is always a tension between the resources needed to perform the best possible experiment and the resources available. The experimental guidelines (Table 1) are designed to minimize the burden on researchers while setting a minimum baseline for rigor to ensure that the field of oligonucleotide research and medicine remains strong.
